# Anesthetic Management and 30-Day Outcomes After Renal Autotransplantation

**DOI:** 10.31486/toj.19.0086

**Published:** 2020

**Authors:** Rovnat Babazade, Jagan Devarajan, Anthony S. Bonavia, Youssef Saweris, Jerome O’Hara, Rafi Avitsian, Hesham Elsharkawy

**Affiliations:** ^1^ Department of Anesthesiology, The University of Texas Medical Branch, Galveston, TX and Outcomes Research Consortium, Anesthesiology Institute, Cleveland Clinic, Cleveland, OH; ^2^ Department of Regional Practice Anesthesiology, Anesthesiology Institute, Cleveland Clinic, Cleveland, OH; ^3^ Department of Anesthesiology and Perioperative Medicine, Penn State Health Milton S. Hershey Medical Center, Hershey, PA; ^4^ Division of Pain Medicine, University Hospitals, Case Medical Center, Cleveland, OH

**Keywords:** *Anesthesia–general*, *kidney transplantation*, *renal autotransplant*

## Abstract

**Background:** Renal autotransplantation is a complex procedure performed for various indications such as treatment of renal vascular and urologic lesions and loin pain hematuria syndrome (LPHS). Because of the rarity of the procedure, few reports have been published, and little is known about anesthetic management and postoperative outcomes of patients with LPHS. The goal of this study was to review and describe all cases of renal autotransplantation performed at Cleveland Clinic during a specified period, focusing on anesthetic management and postoperative 30-day outcomes.

**Methods:** We performed a retrospective review of the records of all patients who underwent renal autotransplantation from 2005 to 2014 at the Cleveland Clinic and collected demographic, anesthetic, surgical, and postoperative data.

**Results:** A total of 64 patients underwent renal autotransplantation from 2005 to 2014. The most frequent indications were nephrolithiasis and LPHS. General endotracheal anesthesia with epidural for pain control was used in 47% of cases. Median duration of anesthesia was 528 minutes. Most patients were sent to a regular nursing floor postoperatively, but 28% of patients required intensive care unit admission. Two patients developed graft ischemia, and 1 patient developed graft failure requiring nephrectomy. No anesthetic-related complications and no mortality were associated with this procedure during the study.

**Conclusion:** Renal autotransplantation is a safe option for patients with LPHS. Additional studies are needed to assess the effect of intraoperative anesthetic management on outcomes in this patient population.

## INTRODUCTION

Renal autotransplantation is a complex procedure performed for various indications,^1^ including the correction of renal vascular abnormalities and the management of intractable loin pain hematuria syndrome (LPHS).^1,2^ Autotransplantation was first performed in 1982 for the treatment of LPHS.^3^

In patients with LPHS, the pain is located in the flank region and is often progressive and crippling.^4^ Available treatment options include conservative medical management, psychological evaluation–psychotherapy,^5,6^ nerve blocks, invasive selective renal denervation, and renal autotransplantation.^7,8^ While renal autotransplantation is considered a last resort for the treatment of LPHS, it is the only procedure associated with long-term success, reported to be as high as 75%.^8,9^

Renal autotransplantation involves a laparotomy incision and explantation of one of the kidneys, followed by reimplantation at a different site.^1^ In addition to bleeding and infection, the procedure is associated with rare

complications such as graft dysfunction and recurrence of pain.^10^ Anesthetic management for renal autotransplantation can be challenging, and anesthesiologists play a critical role in preserving renal function during periods of ischemia and reperfusion.^11^ The literature regarding anesthetic management of these patients is scarce, particularly for patients undergoing renal autotransplantation for the treatment of LPHS.^12-14^ In this article, we report our single-institution experience with perioperative anesthetic management of renal autotransplantation and analyze the 30-day outcomes following surgery.

## METHODS

After receiving approval from the Cleveland Clinic Institutional Review Board (IRB# 15-132), we conducted a retrospective medical record review, searching our surgical database for the keywords “renal autotransplantation.” The search identified 64 patients during the 9-year period of 2005 to 2014. The investigators reviewed the electronic medical records and anesthesia charts for each patient, capturing demographic and personal health information, including comorbid medical conditions, as well as intraoperative and postoperative variables for each patient. Information about postoperative complications in the 30 days following surgery was also collected, including graft rejection, acute kidney injury (AKI), ileus, and infection. Surgical duration was defined as the time interval between skin incision and completion of wound closure. Graft failure was defined as functional failure of the transplanted kidney and development of AKI following surgery. Sepsis was defined as systemic inflammatory response syndrome in response to an infectious process. For patients admitted to the intensive care unit (ICU) postoperatively, the nature of the complication resulting in ICU admission and any treatment received were analyzed. Data regarding patient length of hospital stay were also collected.

## RESULTS

Demographic and preoperative characteristics of the 64 patients who underwent renal autotransplantation during the 9-year study period are presented in Table 1. The median age was 40.1 years, and sex distribution was 36% males and 64% females. Thirty-five (55%) patients had an American Society of Anesthesiologists (ASA) classification of III, and 20 (31%) patients were classified as ASA II. Seventy-two percent of patients were on chronic opioid therapy in the preoperative period.

**Table 1.** Demographics and Preoperative Characteristics (n=64)

Variable	Value
Median age, years (range)	
All patients	40.1 (16-72)
Female	39.3 (16-64)
Male	41.6 (23-72)
Median weight, kg (range)	79 (41-136)
Sex	
Female	41 (64)
Male	23 (36)
American Society of Anesthesiologists classification	
I	2 (3)
II	20 (31)
III	35 (55)
IV	1 (2)
Missing	6 (9)
Indication	
Recurrent nephrolithiasis/obstruction	29 (45)
Loin pain hematuria syndrome	16 (25)
Hydronephrosis/ureteral stricture	9 (14)
Renal artery aneurysm	7 (11)
Renal artery dysplasia	3 (5)
Comorbidity	
Coronary artery disease	4 (6)
Hypertension	23 (36)
Diabetes mellitus	2 (3)
Asthma	7 (11)
Cerebrovascular accident	2 (3)
Chronic pain medications	46 (72)

Note: Data are presented as n (%) unless otherwise indicated.

Most of the patients (29, 45%) were autotransplanted to relieve ureteral obstruction. Among them, 25 had nephrolithiasis, and 2 patients each had retroperitoneal fibrosis and desmoid tumors causing obstructive uropathy. Sixteen (25%) patients had chronic LPHS. The remaining patients had renal artery aneurysm, hydronephrosis/ureteral stricture, or renal artery dysplasia.

Intraoperative data are presented in Table 2. In addition to standard ASA monitoring and routine intraarterial blood pressure monitoring in all the patients, 10 patients had central venous pressure monitoring as well. Median surgical duration was 435 minutes. All patients had general endotracheal anesthesia with intravenous (IV) induction (propofol and fentanyl) and general anesthesia maintained with volatile anesthetic (sevoflurane or isoflurane 1.0 to 1.5 minimum alveolar concentration) throughout the surgery. Thirty (47%) patients received an epidural for postoperative analgesia. The epidural catheter was placed preoperatively and commonly started intraoperatively. The epidural solution contained fentanyl 2 mcg/mL and bupivacaine 0.1%, given at a basal rate of 5 to 7 mL/h, with demand boluses of 6 mL and a lockout interval of 15 minutes. Rescue analgesia was given per hospital policy (ordered by clinicians if the patient reported a pain score on the numeric rating scale ≥4) and included opioids.

**Table 2.** Intraoperative Characteristics (n=64)

Variable	Value
Open surgery	64 (100)
General anesthesia	64 (100)
Combined general anesthesia/epidural analgesia	30 (47)
Central venous pressure monitoring	10 (16)
Median duration of surgery, min (range)	435 (53-800)
Median duration of anesthesia, min (range)	528 (103-937)
Renal protection	
None	2 (3)
Mannitol 12.5 g	18 (28)
Mannitol 25 g	27 (42)
Mannitol >25 g	6 (9)
Missing	11 (17)
Median estimated blood loss, mL (range)	1,053 (120-3,000)
Blood transfusion	29 (45)
Median crystalloid administration, mL (range)	7,144 (500-17,200)
Median colloid administration, mL (range)	1,253 (0-3,500)
Median urine output, mL (range)	2,003 (415-4,950)

Note: Data are presented as n (%) unless otherwise indicated.

Every effort was made to provide adequate renal perfusion, which involved maintenance of euvolemia and adequate perfusion pressures. A median of 7,144 mL of crystalloids and a median of 1,253 mL of colloids were administered during the procedure. Fifteen patients received both colloid and mannitol. Median blood loss was 1,053 mL, and 29 patients required blood transfusion.

Postoperative and 30-day outcomes data are presented in Table 3. The median length of stay was 8 days, with 18 patients requiring ICU admission. The indications for ICU admission included severe sepsis, AKI, ileus, lower left extremity weakness, atrial fibrillation, anemia, and adult respiratory distress syndrome. Most patients with LPHS (75%) had a clinically significant reduction in pain as assessed by their 30-day postoperative period pain scores, but 4 (25%) patients with LPHS had less-than-optimal pain relief following surgery.

**Table 3.** Postoperative and 30-Day Outcomes (n=64)

Assessment Period	Outcome	Value
In hospital postoperatively	Median length of hospital stay, days (range)	8 (5-26)
	Intensive care unit admission	18 (28)
	Complications	
	None	32 (50)
	Ileus	8 (13)
	Acute kidney injury	5 (8)
	Sepsis	4 (6)
	Anemia	4 (6)
	Lower left extremity pain/weakness	2 (3)
	Graft ischemia	2 (3)
	Nausea	1 (2)
	Renal ischemia	1 (2)
	Adult respiratory distress syndrome	1 (2)
	Anxiety	1 (2)
	Atrial fibrillation	1 (2)
	Postoperative pain	1 (2)
	Insomnia	1 (2)
Discharge to 30 days postoperatively	Pain	
	None/resolved pain	54 (84)
	Improved pain	6 (9)
	Persistent/recurrent pain	4 (6)
	Loin pain hematuria syndrome–related pain (n=16)	
	None/resolved pain	12 (75)
	Improved pain	0 (0)
	Persistent/recurrent pain	4 (25)
	Complications	
	Nephrectomy	3 (5)
	Small bowel resection	1 (2)
	Graft failure	1 (2)
	Anesthesia-related	0 (0)
	Death	0 (0)

Note: Data are presented as n (%) unless otherwise indicated.

The 7 patients who underwent surgery for renal artery aneurysm had an uneventful perioperative course. A patient with bilateral desmoid tumors underwent bilateral autotransplantation in 2 separate operations. The patient had a successful autotransplantation on one side but developed graft failure on the second side and required nephrectomy. The patient developed sepsis and required ICU admission.

Despite prolonged surgery, all patients were successfully extubated in the operating room immediately following the procedure. Most patients were sent to a regular nursing floor postoperatively. Two patients developed graft ischemia, requiring nephrectomy. None of the patients developed postoperative renal failure requiring hemodialysis. None of the patients developed hemodynamic instability that necessitated vasopressor infusion therapy intraoperatively. No mortality was associated with this procedure during the study period.

## DISCUSSION

To our knowledge, this study is the largest case series reporting anesthetic management of patients undergoing renal autotransplantation in the English-language literature,^13,15^ with the largest subset of autotransplantation procedures performed for a diagnosis of LPHS.

Renal autotransplantation is a highly invasive surgery that is usually performed for benign indications.^16^ Common indications include renal artery stenosis causing renovascular hypertension,^17^ correction of large aneurysms or arteriovenous fistulae, and repair of complex ureteral lesions. Renal autotransplantation is occasionally performed to treat centrally located renal tumors or multiple intrarenal tumors of a solitary kidney. As previously stated, the procedure is also used to treat LPHS, and long-term success rates have been reported as high as 75%.^8,9^

The surgery involves excision of the nerves around the renal hilum, resulting in near-complete denervation of the kidney.^18^ Denervation of the kidney interrupts transmission of nociceptive signals from the kidney and ureter, thereby resolving pain. The characteristic pain of LPHS usually disappears soon after surgery in the majority of patients, allowing them to be weaned off their preoperative opioid medications. However, pain can reoccur at the graft site if reinnervation of the renal hilum and ureter occurs.

LPHS is usually diagnosed in middle-aged healthy adults. In one study, the mean age was 41 years (range, 34 to 59 years), and none of the patients had any major or intermediate preoperative cardiac risk factors.^19^ The literature indicates a propensity of LPHS to affect females more commonly than males.^8^ In our study, the median age was 40.1 years, and 64% of patients were female, results that corroborate the literature.

The recurrence of pain after autotransplantation has been attributed to reinnervation through autonomic nerves carrying nociceptive impulses.^20^ In the review by Dewar and Chin, the majority of patients undergoing the procedure had chronic pain caused by either LPHS or renal anomalies.^21^ Similarly, the majority of patients (46/64, 72%) in our study also had chronic pain attributed to either LPHS or recurrent nephrolithiasis. A long-term follow-up of the treatment of LPHS with autotransplantation revealed that 75% (12/16) of patients were pain-free for more than 5 years following the procedure.^9^

Because of the high number of patients on preoperative opiates and the requirement for a relatively large laparotomy incision, an epidural is a good option for postoperative analgesia. In our study, 47% of patients received epidural analgesia during the perioperative period. During the 30-day follow-up period, our analgesia success rate was 75% (12/16) in patients who had LPHS, similar to previously reported results.^9^ However, because of the small number of patients, we cannot draw any conclusions about whether epidural analgesia could have played a role in improving long-term analgesic outcomes. Among the 4 patients with LPHS who had persistent pain 30 days after discharge, 2 had received epidural analgesia.

Placement of an epidural catheter is not always possible because of anatomic difficulties or anticoagulation that contraindicates insertion.^22^ Erector spinae plane block via catheter placement for continuous infusion may be warranted given that many patients are anticoagulated as a component of conservative management.

Our operative times were comparable to those in 2 published case series studies—one from the Japanese literature (1998) and one from Portugal (2018).^13,15^ In the case series from Portugal, the median duration of anesthesia for renal autotransplantation surgery was 438 minutes.^15^ In our study, the median surgery and anesthesia durations were 435 and 528 minutes, respectively.

Blood loss depends on many factors such as the complexity of the dissection, the presence of collaterals from the aorta and vena cava, and a history of previous abdominal surgeries. In contrast to the case series from Portugal,^15^ our patients’ intraoperative estimated blood loss was increased by almost 2 times, and our patients required 3 times more blood and fluid transfusions. The median estimated blood loss in our patients was 1,053 mL with 29 (45%) patients requiring blood transfusion during the intraoperative period.

AKI prevention is a vital component of the perioperative management of patients undergoing renal autotransplantation, and anesthesiologists play a crucial role in preventing such injury. Maintaining perioperative euvolemia and adequate renal perfusion have consistently been shown to prevent AKI.^23^ IV fenoldopam 0.1 mcg/kg/min after induction of anesthesia has been shown to reduce the incidence of AKI, the renal replacement therapy requirement, and mortality.^24,25^ Although major studies of patients undergoing autotransplantation have not been done, the results from studies about patients undergoing allogenic renal transplantation can be applied to this procedure.^24,25^

In our patients, conservative medical management with anticoagulants, antiplatelets, and long-term antibiotics failed to provide relief for LPHS. Percutaneous splanchnic nerve blockade, topical injection of capsaicin and bupivacaine, and celiac plexus block have also been tried without much long-term success.^26^ Surgical renal denervation without autotransplantation was complicated by high rates of recurrence.^27^ Other procedures that have been used include different combinations of perivascular stripping of nerve plexuses from the kidney and capsulotomy that resulted in temporary—at the most a few months—pain relief.^18,28^ In one case series, the pain was so severe that it led to nephrectomy in 38% of patients with LPHS.^27^

Major complications from this procedure are rare. In our series, 4 patients developed sepsis during postoperative period, and another patient experienced graft failure. None of the cases involved intraoperative complications or anesthetic-related postoperative complications. Sevmis et al did not report any morbidity and mortality associated with the procedure.^29^ Webster et al reported few complications.^30^ In the Webster et al series, 1 patient had a suture abscess, and another had chronic wound pain. One patient each developed acute tubular necrosis and multisystem organ failure.

Tran et al performed laparoscopic nephrectomy with autotransplantation on 52 patients, and their long-term (>6-year median follow-up) experience validates the efficacy of the procedure (>90% preserved renal unit function) with an acceptable incidence (12%) of late major complications.^31^ Five (10%) patients had autotransplanted renal losses at a median of 15 months, and 4 of them required autotransplant nephrectomy.^31^ In our study, 3 (5%) patients—2 patients with graft ischemia and 1 patient with graft failure—required nephrectomy. We did not have long-term follow-up and therefore cannot comment on the risk of subsequent nephrectomy. Despite the potential complications, the strong ethical argument in favor of autotransplantation is that this procedure offers the best possible chance for long-lasting pain relief with the highest likelihood of retaining renal function.

As with all retrospective studies, our study has many limitations. Long-term follow-up is lacking, so we have no data on any complications that occurred after the 30-day follow-up period. Our study does not address whether pain relief from the procedure translated to improved social, economic, and emotional outcomes. Our data are from a single tertiary care center with considerable resources and experience in urologic, transplant, pain management, and vascular surgery. A multidisciplinary approach with a knowledgeable and experienced team is necessary to the success of renal autotransplantation. Therefore, this study may not be generalizable to some academic institutions and community hospitals.

## CONCLUSION

Renal autotransplantation is one of the most effective therapeutic options for management of patients with LPHS. Anesthesiologists play a critical role in minimizing renal insult during and following this major abdominal surgery. Further studies are needed to determine whether goal-directed fluid and multimodal pain management influence postoperative outcomes in these patients.

## Notes

This article meets the Accreditation Council for Graduate Medical Education and the American Board of Medical Specialties Maintenance of Certification competencies for Patient Care and Medical Knowledge.
